# BMI, race and AF recurrence post ablation: a meta-analysis and systematic review

**DOI:** 10.3389/fcvm.2026.1434751

**Published:** 2026-04-08

**Authors:** Ye Xuejiao, Lv Qianyu, Hou Xinzheng, Lv Yanfei, Li Lanlan, Yang Yingtian, Yang Chenyan, Wang Manshi, Li Yushan, Chang Xing, Wu Qian, Wang Shihan

**Affiliations:** 1 Cardiovascular Department, China Academy of Chinese Medical Sciences, Guang'anmen Hospital, Beijing, China; 2 School of Management, Fudan University, Shanghai, China; 3 School of Chinese Medicine, Beijing University of Chinese Medicine, Beijing, China; 4 Department of Cardiology, Beijing Xicheng District Guang‘wai Hospital, Beijing, China

**Keywords:** atrial fibrillation, body mass index, obesity, race, systematic review and meta-analysis

## Abstract

**Aims:**

Obesity increases atrial fibrillation (AF) risk, but it remains unclear whether this association can be extrapolated to AF patients who undergoing pulmonary vein isolation (PVI), and whether there are racial differences. This study aims to explore the role and interaction of body mass index (BMI) and racial factors in the risk of AF recurrence post PVI.

**Methods:**

A systematic search was conducted in PubMed, Web of Science, Engineering Index, Wan Fang database, CNKI, and relevant meta-analyses from database inception to May 19, 2025. Standardized data extraction was performed in accordance with the Meta-analysis of Observational Studies in Epidemiology Reporting Guidelines. Primary outcome was the recurrence of AF post PVI. Secondary outcomes included procedural complications, and echocardiographic parameters.

**Results:**

Finally, 26 studies involving 35,237 individuals were included. The results showed a linear positive correlation between BMI and the risk of AF recurrence post PVI. For every 5-units increase in BMI, the recurrence risk of AF increased by 9%. Subgroup analysis indicated that obese Europeans had a 9% higher recurrence risk (*P* < 0.001), obese North Americans had a 14% higher recurrence risk (*P* < 0.001), and Asians had the highest increase in recurrence risk, reaching 37% (*P* < 0.001). Meta-regression analysis suggested that racial differences were potentially related to the strength of the association between BMI and AF recurrence (*P* = 0.033). Risk factor analysis identified enlarged left atrial diameter as a potential risk factor contributing to the recurrence of AF (*P* = 0.021). No statistically significant difference in surgical complications risk was observed between obese and non-obese populations (*P* = 0.083).

**Conclusion:**

Our findings confirm a linear positive correlation between BMI and the risk of AF recurrence post PVI. An enlarged left atrial diameter may be one potential mechanisms underlying AF recurrence. Furthermore, significant racial disparities were identified in AF recurrence risk, with Asian patients showing a significantly higher recurrence risk than European and American patients.

**Systematic Review Registration:**

https://www.crd.york.ac.uk/prospero/display_record.php?ID=CRD42024496463, PROSPERO CRD42024496463.

## 1 Introduction

Atrial fibrillation (AF) is one of the most common tachyarrhythmias in clinical practice. It was first medically recorded in the early 20th century with the emergence of electrocardiograms (ECG) (1). Current epidemiological evidence shows that AF has gradually become a major challenge in global public health (2). As of 2020, the global prevalence of AF has reached approximately 375 million (3). It is worth noting that studies have shown significant differences in the epidemiological characteristics of AF among different countries, which are influenced by factors such as racial genetic background and geographical environment (4).

Obesity is an independent and modifiable risk factor for AF, with multidimensional pathogenic mechanisms underlying AF development (5–7). Previous studies have confirmed that obesity increases the incidence of new-onset AF by approximately 60% (8). Large-scale cohort studies, including the Framingham Heart Study (FHS), have identified obesity as an independent predictor of AF (5–7, 9). For every 1 unit increase in body mass index (BMI), the risk of AF increases by 4% (10–12). Short-term rapid weight gain significantly increases AF risk, while after weight returns to normal, the risk of AF can significantly decrease (11, 13). Additionally, some well- known AF risk factors, such as hypertension (HTN), diabetes mellitus (DM), are associated with obesity (13, 14). Over the past 50 years, the global prevalence of obesity has doubled and continues to rise. It is predicted that the incidence of AF will further increase in the future (15).

Pulmonary vein isolation (PVI), as first-line therapy for AF, is significantly superior to antiarrhythmic drugs (AADs) in improving AF symptoms and patients' quality of life (16). Clinical data shows that approximately 75% of AF patients can maintain sinus rhythm after ablation (17). However, current research has not fully clarified whether obesity affects PVI efficacy or if there are racial differences in this effect. Given this research gap, we conducted this systematic meta-analysis to evaluate the clinical role of BMI and racial factors in PVI treatment.

## 2 Methods

This systematic review and meta-analysis was performed in accordance with the Meta-analysis of Observational Studies in Epidemiology (MOOSE) Reporting Guidelines(Supplementary MOOSE Checklist.pdf) (18). The protocol was registered on the International Prospective Register of Systematic Reviews (PROSPERO) network (Registration number: CRD42024496463).

### 2.1 Search strategy and study selection

Two authors (YX and WQ) independently conducted a systematic literature search in PubMed, Web of Science, Embase, Engineering Index, Wan Fang database, and CNKI from inception to May 19, 2025. The search strategy used MeSH terms and free words: (BMI) OR (body mass index) OR (obesity) OR (overweight) AND (AF) OR (atrial fibrillation) OR (Auricular fibrillation) AND (ablation) OR (cardiac ablation) OR (radiofrequency ablation) OR (catheter ablation) OR (pulmonary vein isolation surgery) OR (cryoballoon ablation) OR (PVI) OR (CA) OR (RFA), which were combined using the Boolean operator “AND”. Only human studies were eligible, with no restrictions on language or data. Duplicates were removed using EndNote 20 Software. Additionally, we screened the references of relevant literature and previously published meta-analyses (19–22) to identify other potential studies that were not covered in the initial database searches. For studies with duplicate samples data, the one with more comprehensive information will be included. Two authors independently evaluated the literature based on pre-established inclusion and exclusion criteria. Studies for which the full text cannot be obtained were excluded, and any discrepancies will be resolved through discussion or with the involvement of a third party. Taking PubMed as an example, Table 1 presents the full retrieval strategy. The search strategies for other databases can be found in the Supplementary Material.

**Table 1:** Search strategy for PubMed.

PubMed search strategy steps
**Body mass index [MeSH]**
1. ((“Body Mass Index”[MeSH]) OR (“Obese”[MeSH]) OR (“Overweight”[MeSH]) OR (“BMI”[Title/Abstract]) OR (“body mass index”[Title/Abstract]) OR (“obesity”[Title/Abstract]) OR (“overweight”[Title/Abstract]))
**Atrial fibrillation [MeSH]**
2. (“Atrial Fibrillation”[MeSH]) OR (“AF”[Title/Abstract]) OR (“atrial fibrillation”[Title/Abstract]) OR (“auricular fibrillation”[Title/Abstract])
**Catheter Ablation [MeSH]**
3. ((“Catheter Ablation”[MeSH]) OR (“Radiofrequency Ablation”[MeSH]) OR (“Cryoballoon Ablation”[MeSH]) OR (“Pulmonary Vein Isolation”[MeSH]) OR (ablation[Title/Abstract] AND cardiac[Title/Abstract]) OR (PVI[Title/Abstract]) OR (CA[Title/Abstract]) OR (RFA[Title/Abstract]))
4. ((“Humans”[MeSH]) AND (“Clinical Trial”[Publication Type] OR “Observational Study”[Publication Type] OR “Randomized Controlled Trial”[Publication Type]))
5. #1 AND #2 AND #3 AND #4

### 2.2 Eligibility criteria

Randomized controlled trials (RCTs) and cohort studies were included in this meta-analysis if they meet the following criteria:

1. The included AF patients underwent PVI, including radiofrequency ablation, cryballoon ablation, etc.
2. The number of AF recurrences post ablation was clarified in different BMI stratifications.
3. Clear definitions of non-obesity and obesity were provided: obesity was defined as BMI≥28 kg/m^2^ for Asians, and BMI≥30 kg/m^2^ for non-Asians.
4. A blanking period≥3 months and a follow-up duration≥12 months were required.
5. AF recurrence was detected by 12-lead ECG or 24 h Holter monitoring.

Exclusion criteria include reviews, letters, editorials, comments, case reports, low-quality studies with sample sizes <50 or NOS scores≤5, and studies that did not report primary or interested outcomes.

### 2.3 Data extraction

Two investigators (WQ and LY) independently extracted data based on inclusion and exclusion criteria, and consistency of the extraction results was evaluated using a consistency test (Kappa values). Any discrepancies were resolved through discussion or third-party involvement (WS). Duplicate literature was identified and eliminated using the literature management tool EndNote X9. For studies containing multiple subgroups, only summary data or data with clearly labeled sources were included to avoid sample duplication. A standardized data extraction form was used, which included the following items: first author's name, publication year, study design, sample size, country, geographic region, follow-up duration, blanking period, endpoint evaluation method, AF recurrence rate, surgical complications, baseline patient characteristics (such as age, gender, race, AF duration, comorbidity, LAD and LVEF), ablation strategy, and AF type. The detailed standardized extraction form was provided in the Supplementary File S1: Standardized data extraction form 1.0.xlsx. The primary outcome was the recurrence of AF, which was detected by 12-lead ECG or 24 h Holter monitoring. The secondary outcomes included surgical complications, patients' comorbidity such as HTN, DM, and other relevant indicators such as LAD and LVEF.

Standardized extraction form included:

1. Basic information of included literature: First author's name, publication year, study design, sample size, country, geographic region, and follow-up duration.
2. Baseline characteristics of samples: Age, gender, race, comorbidity such as HTN, DM, and other relevant indicators such as LAD, LVEF, duration of AF.
3. Primary outcomes: Number of AF recurrence in different BMI stratifications.
4. Secondary outcomes: Surgical complications, patients' comorbidity such as HTN, DM, and other relevant indicators such as LAD, LVEF.

### 2.4 Quality assessments

The methodological quality of included studies was independently assessed by two authors (YX and LQ) using the Newcastle-Ottawa Scale (NOS) (23). Any discrepancies were resolved through discussion. The validated NOS comprises 3 major components: 1) selection of patients (scored from 0 to 4 stars), 2) the comparability of cohorts (scored from 0 to 2 stars), 3) ascertainment of the outcome (scored from 0 to 3stars). Studies with a NOS score≥5 stars were classified as moderate to high quality. Conversely, studies with a score <5 stars were considered low-quality (24).

### 2.5 Statistical analysis

All statistical analyses were performed using Review Manager 5.3 (Rev Man 5.3) and Stata 14.0 (Stata Crop LLC, Texas, US). For categorical variables, risk ratio (RR) with 95% confidence interval (95% CI) were calculated. For continuous variables, mean difference ± standard mean deviation (MD ± SMD) with 95% CI were used as statistical indicators. Statistical significance was defined for results with *P* < 0.05. Statistical heterogeneity among studies was evaluated using Cochran's *Q* test and *I^2^* statistic. If *I^2^*>50% and *P* < 0.10, it indicates the existence of heterogeneity, and a random-effects model was used. Otherwise, a fixed-effects model was applied. If necessary, subgroup analysis will be performed to identify the sources of heterogeneity. Funnel plot analysis and Egger's regression analysis were conducted to assess publication bias and small-study effects, with *P* < 0.05 indicating the existence of publication bias. Meta-regression analysis was performed based on study design, race, ablation strategy, follow-up duration, and sample size to explore potential associations and factors underlying inter-studies differences.

## 3 Results

### 3.1 Study selection

**Figure 1:**
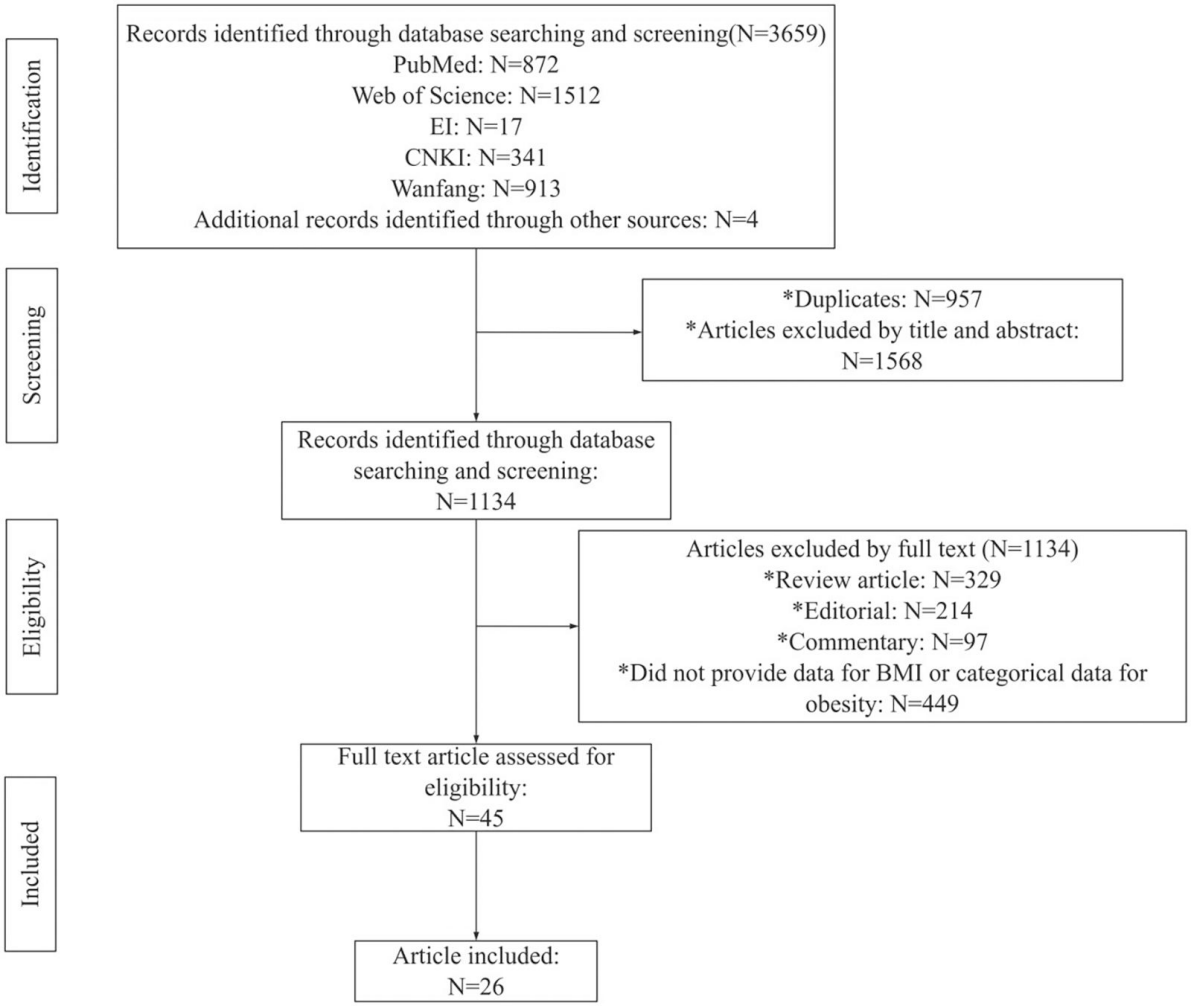
Flowchart of study selectio.

A total of 3,659 records were screened in the initial search. After removing duplicates, 2,702 records remained. Subsequently, 1,568 records were further excluded by title/abstract screening, leaving 1,134 records for full-text analysis. Among the 1,134 articles, 1,089 were excluded for the following reasons: 1) Review (*n* = 329); 2) Editorials (*n* = 214); 3) Commentaries (*n* = 97); 4) Failure to provide BMI-related data or categorical obesity data (*n* = 449). Eventually, 26 studies (5, 7, 25–37), involving a total of 35,237 individuals, met the inclusion criteria. Among them, 8 studies included North Americans from the USA and Canada, 5 included Asians from China, 12 included Europeans from Germany, Italy, France, and the Netherlands, and 1study included a mixed population of Europeans and Americans. Of the 26 selected studies, 9 were prospective cohort studies and 17 were retrospective cohort studies, with follow-up duration ranging from 12 to 48 months. The detailed study selection process was shown in Figure 1.

### 3.2 Baseline characteristics for enrolled studies

A total of 35,237 individuals were enrolled in the 26 studies. The proportions of obese individuals and male participants were 32.8% and 68.1%, respectively. The sample size of the included studies ranged from 87 to 9,188, with a mean age of 62.0 ± 10.7 years. Paroxysmal AF accounted for 43.9% of cases. All studies adopted a 3-months blanking period, and all participants were followed-up and monitored using ECG or 24 h Holter. The baseline characteristics of the included studies and participants are presented in Table 2.

**Table 2:** Baseline characteristics for enrolled studies.

Author (Year)	Country	Region	Design	Sample size (n)	Age (Y)	Male (*n*, %)	Strategy	Blank Period (mos)	Follow-up (mos)	PAF (*n*, %)
Boehmer (32)	Germany	Europe	PC	949	69.1 ± 10.5	540 (56.9)	CBA	3	12	566 (59.6)
Blockhaus (33)	Germany	Europe	RC	228	60.8 ± 10.0	160 (70.2)	CBA	3	12	160 (70)
Bunch (34)	USA	North America	RC	1,558	65.1 ± 10.6	943 (60.5)	PVI	3	12	887 (57)
Calero (35)	Spain	Europe	RC	114	54.5 ± 9.6	86 (75.4)	PVI	3	12	80 (70)
Cha (28)	China	Asia	PC	523	54 ± 10	439 (84)	RFA	3	12	301 (58)
Chilukuri (29)	USA	North America	PC	109	60 ± 10	85 (78)	RFA	3	12	74 (68)
Deng (7)	China	Asia	PC	1,410	57.2 ± 11.6	959 (68)	PVI	3	12	1,090 (77.3)
Glover (36)	Canada	North America	PC	3,333	57.9 ± 10.4	2,268 (68)	PVI	3	12	2,239 (67.2)
Khair (27)	Sweden	Europe	RC	87	62.6 ± 8.7	65 (74.7)	PVI	3	12	NR
Papathanasiou (30)	Greece	Europe	PC	85	60 ± 10	51 (60)	CBA	3	12	85 (100)
Lakkireddy (37)	USA/France	Europe	PC	854	55.1 ± 10.9	676 (79.2)	PVI	3	12	469 (55)
Letsas (50)	Germany	Europe	RC	226	55.9 ± 9.6	184 (81.4)	PVI	3	12	134 (59.3)
Malaspina (51)	Italy	Europe	PC	2,048	59.8 ± 10.6	1,481 (72.3)	CBA	3	12	1,528 (74.6)
Kong (52)	USA	North America	RC	217	64.2 (24,89)	148 (68.2)	PVI	3	12	NR
Providência (53)	France	Europe	PC	2,497	61.1 ± 10.2	1,763 (70.6)	PVI	3	12	1,438 (57.6)
Sivasambu (5)	USA	North America	RC	701	59.7 ± 10.0	504 (71.9)	PVI	3	12	416 (59.3)
Tabaja (54)	USA	North America	RC	5,841	64.4 ± 10.1	4,154 (71.1)	PVI	3	12	2,748 (47)
Tang (31)	China	Asia	RC	488	57 ± 11.4	342 (69.4)	RFA	3	12	488 (100)
Tønnesen (55)	Denmark	Europe	RC	9,188	62.9 ± 9.7	6,489 (70.6)	NR	3	12	NR
Urbanek (56)	Germany	Europe	RC	600	66 ± 11	356 (59)	CRB	3	12	348 (58)
Weinmann (57)	Germany	Europe	RC	600	66.3 ± 10.8	340 (57)	CRB	3	12	522 (87)
Winkle (49)	USA	North America	RC	2,715	63.9 ± 10.3	1,887 (69.5)	PVI	3	12	887 (32.7)
Wolfes (58)	Germany	Europe	RC	226	60.1 ± 11.2	141 (62.4)	PVI	3	48	108 (47.8)
Wylie (59)	USA	North America	RC	523	NR	NR	PVI	3	24	303 (58)
Lin (26)	China	Asia	RC	140	60.1 ± 10.4	92 (65.7)	PVI	3	12	140 (100)
Xu (25)	China	Asia	RC	471	66.3 ± 9.0	268(56.9)	PVI	3	20.5	471(100)

PC: Prospective cohort; RC: Retrospective cohort; PAF: Paroxysmal atrial fibrillation. NR: Not report.

### 3.3 Quality assessment

The quality assessment results of the included studies were presented in Table 3. Specifically, 6 studies attained 9 stars, 19 studies obtained 8 stars, and 1 studies reached 7 stars. Overall, the quality of the included studies was deemed acceptable, with all NOS scores were≥7 stars. (Table 3).

**Table 3:** Quality assessment for enrolled studies (Newcastle-Ottawa scale).

Author (Year)	Selection				Comparability		Outcome			Total
	^a^	^b^	^c^	^d^	^e^	^f^	^g^	^h^	^i^	
Boehmer (32)	1	1	1	1	1	1	1	1	1	9
Blockhaus (33)	1	1	1	1	1	1	1	1	1	9
Bunch (34)	1	1	1	1	0	1	1	1	1	8
Calero (35)	1	1	1	1	1	1	0	1	1	8
Cha (28)	1	1	1	1	1	1	0	1	1	8
Chilukuri (29)	1	1	1	1	0	1	1	1	1	8
Deng (7)	1	1	1	1	0	1	1	1	1	8
Glover (36)	1	1	1	1	1	1	1	1	1	9
Khair (27)	1	1	1	1	0	1	1	1	1	8
Papathanasiou (30)	1	1	1	1	0	1	1	1	1	8
Lakkireddy (37)	1	1	1	1	0	1	1	1	1	8
Letsas (50)	1	1	1	1	0	1	0	1	1	7
Malaspina (51)	1	1	1	1	1	0	1	1	1	8
Kong (52)	1	1	1	1	0	1	1	1	1	8
Providência (53)	1	1	1	1	1	1	1	1	1	9
Sivasambu (5)	1	1	1	1	0	1	1	1	1	8
Tabaja (54)	1	1	1	1	0	1	1	1	1	8
Tang (31)	1	1	1	1	1	1	0	1	1	8
Tønnesen (55)	1	1	1	1	1	1	1	1	1	9
Urbanek (56)	1	1	1	1	0	1	1	1	1	8
Weinmann (57)	1	1	1	1	1	1	1	1	1	9
Winkle (49)	1	1	1	1	1	1	0	1	1	8
Wolfes (58)	1	1	1	1	1	1	1	1	0	8
Wylie (59)	1	1	1	1	0	1	1	1	1	8
Lin (26)	1	1	1	1	0	1	1	1	1	8
Xu (25)	1	1	1	1	0	1	1	1	1	8

^a^ Representativeness of exposed cohort.

^b^ Selection of the non-exposed cohort.

^c^ Ascertainment of exposure.

^d^ Demonstration that outcome of interest was not present at start of study.

^e^ Comparability of cohorts on the basis of the design or analysis.

^f^ Comparability of cohorts on the basis of the design or analysis (adjusted for any other factor).

^g^ Assessment of outcome.

^h^ Was follow-up long enough for outcomes to occur. (>=1 years).

^i^ Adequacy of follow-up of cohorts.

### 3.4 Analysis of the impact of BMI on AF recurrence: overall and subgroup

**Figure 2:**
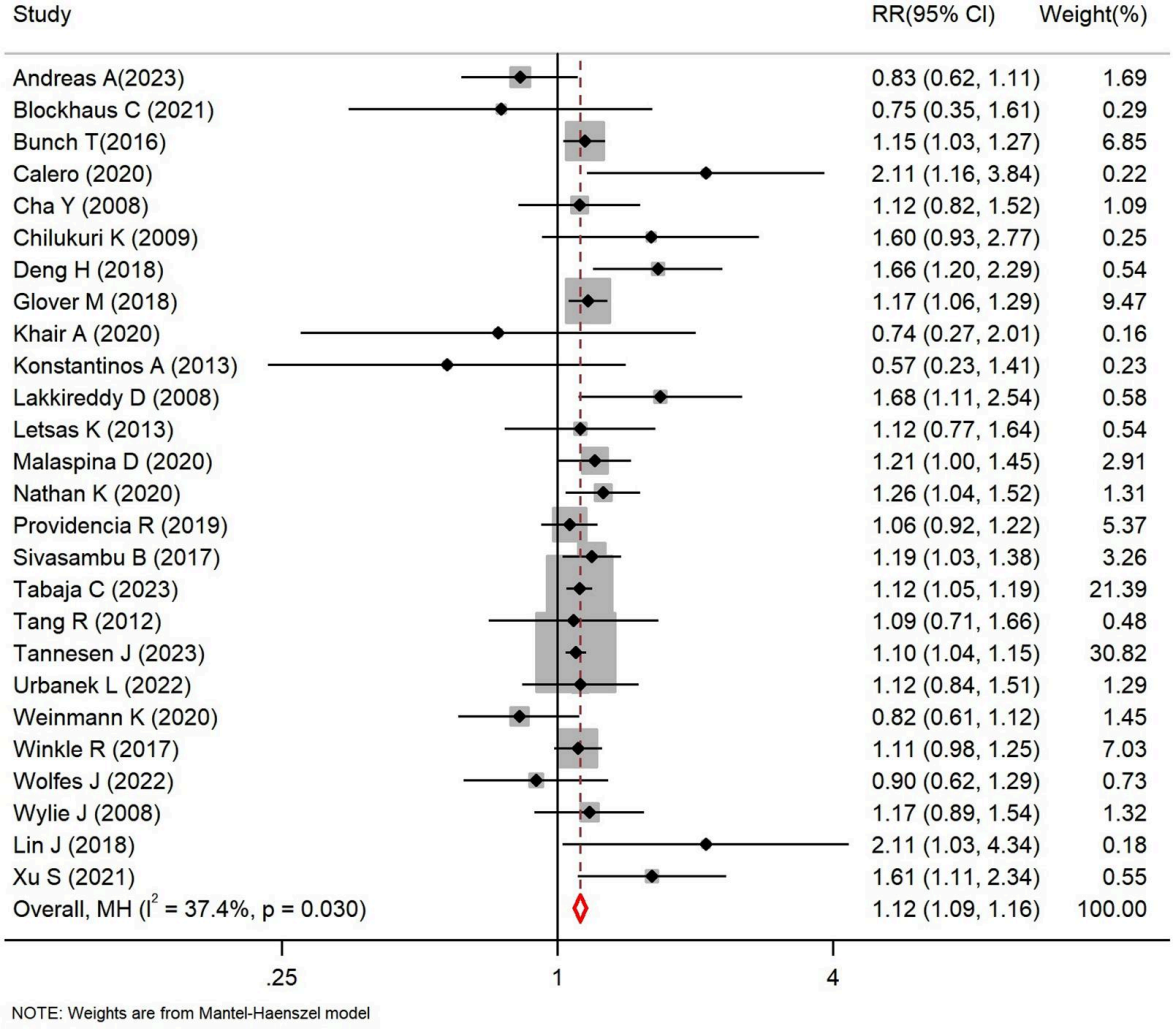
Forest plot of the association between BMI and AF recurrence.

**Figure 3:**
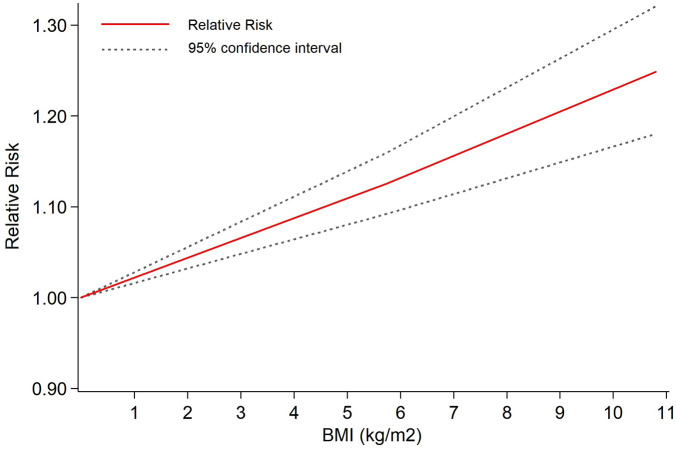
Linear dose-response analysis of BMI-increase-and risk of AF recurrence after ablation. The solid and dashed lines represent the estimated relative risk and the 95% confidence interval, respectively.

The impact of BMI on AF recurrence is shown in Table 4. Overall analysis shows that obesity was significantly associated with the risk of AF recurrence post ablation, with a pooled RR of 1.12 (*P_value_ *< 0.001), indicating that obesity increases the risk of AF recurrence post ablation by 12% (*I^2^* = 37.4%, *P_heterogeneity_* = 0.030, 95% CI: 1.09∼1.16, Figure 2). Dose-response analysis revealed a linear upward trend in the relative risk of AF recurrence with increasing BMI: for every 5 units increase in BMI, the risk of AF recurrence increases by 9%(Figure 3). This suggests that BMI may serve as a predictive indicator for AF recurrence post ablation. Subgroup analysis demonstrated a positive correlation between BMI and AF recurrence in almost all subgroups. Notably, racial differences may be an important potential factor influencing AF recurrence post ablation. Specifically, obese European patients had a 9% higher AF recurrence risk than non-obese individuals (RR = 1.09, *I^2^* = 44.1%, *P_heterogeneity_*=0.044, 95%CI:1.04–1.13, *P_value_ *< 0.001), while obese North American patients had a 14% higher recurrence risk (RR = 1.14, *I^2^* = 0.0%, *P_heterogeneity_* = 0.813, 95% CI:1.09–1.19, *P_value_ *< 0.001). Among these, Asian populations showed the most significant increase in AF recurrence risk (RR = 1.37, *I^2^* = 35.8%, *P_heterogeneity_* = 0.182, 95% CI:1.16–1.63, *P_value_ *< 0.001). These results suggest potential racial heterogeneity in the mechanism of AF recurrence among obese populations across different regions.

**Table 4:** Subgroup analysis of BMI and AF recurrence post ablation.

Items	Outcome	Study(n)	RR	*I^2^*(%)	*P_heterogeneity_*	95% CI	*P_value_*
Overall		26	1.12	37.4	0.030	1.09–1.16	<0.001
Race	Europe	13	1.09	44.1	0.044	1.04–1.13	<0.001
	North America	8	1.14	0.0	0.813	1.09–1.19	<0.001
	Asia	5	1.37	35.8	0.182	1.16–1.63	<0.001
Publish year	Pre-2020	14	1.16	20.7	0.229	1.10–1.21	<0.001
	Post-2020	12	1.10	48.7	0.029	1.06–1.14	<0.001
Sample size	≥500	15	1.12	32.5	0.108	1.08–1.15	<0.001
	<500	11	1.22	41.2	0.074	1.08–1.39	0.002
Ablation strategy	RFA	11	1.17	3.9	0.406	1.09–1.26	<0.001
	CBA	7	1.03	64.9	0.009	0.83–1.28	0.777
	PVI	8	1.13	33.1	0.163	1.07–1.19	<0.001
Design	PC	7	1.12	53.0	0.047	0.97–1.29	0.134
	RC	19	1.15	33.6	0.077	1.09–1.21	<0.001
Follow-up	≥12 mos	23	1.14	36.8	0.041	1.08–1.20	<0.001
	<12 mos	3	1.19	58.4	0.090	0.88–1.61	0.259

### 3.5 Sensitivity analysis and publication bias

**Figure 4:**
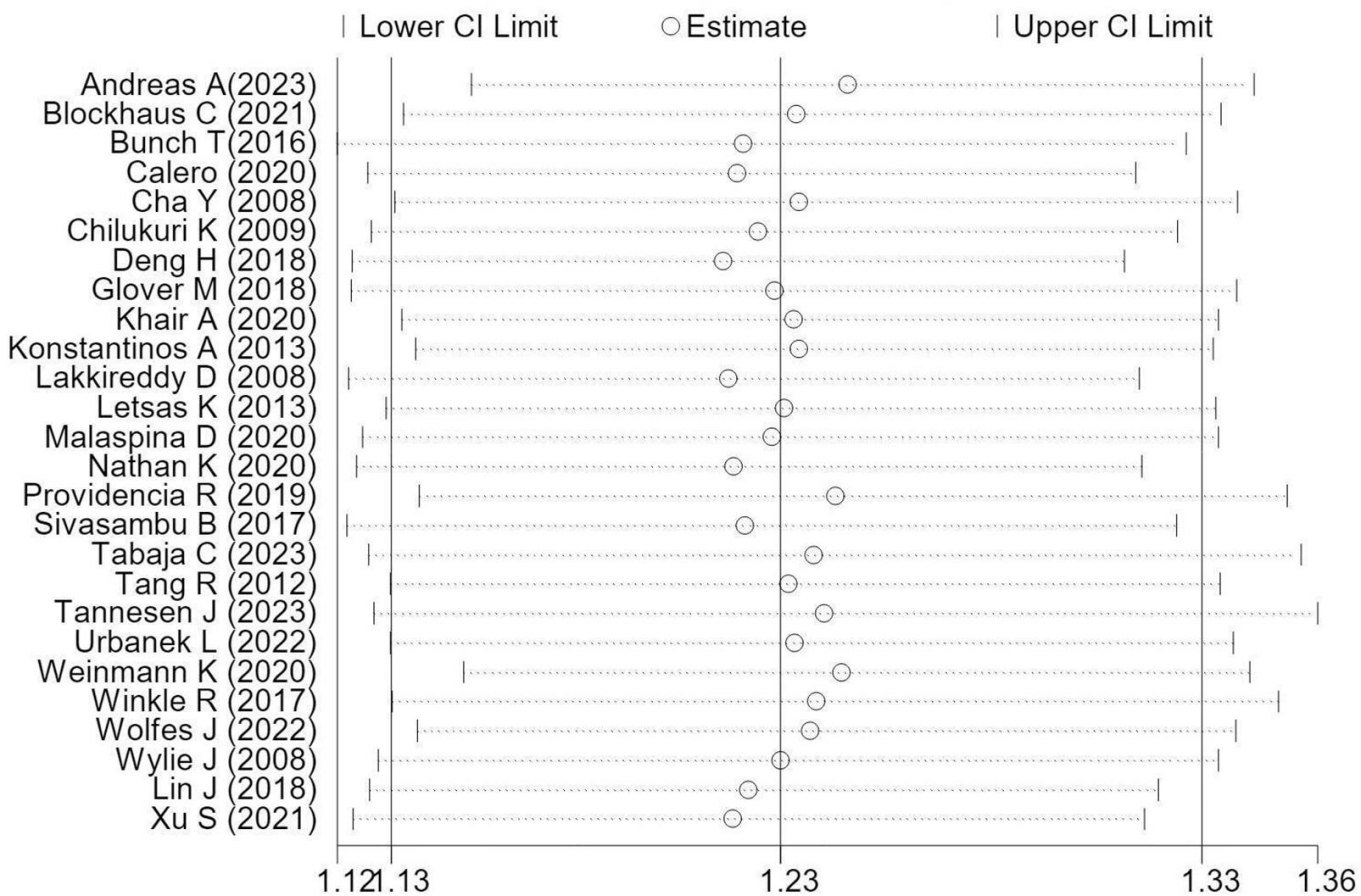
Sensitivity analysis.

**Figure 5:**
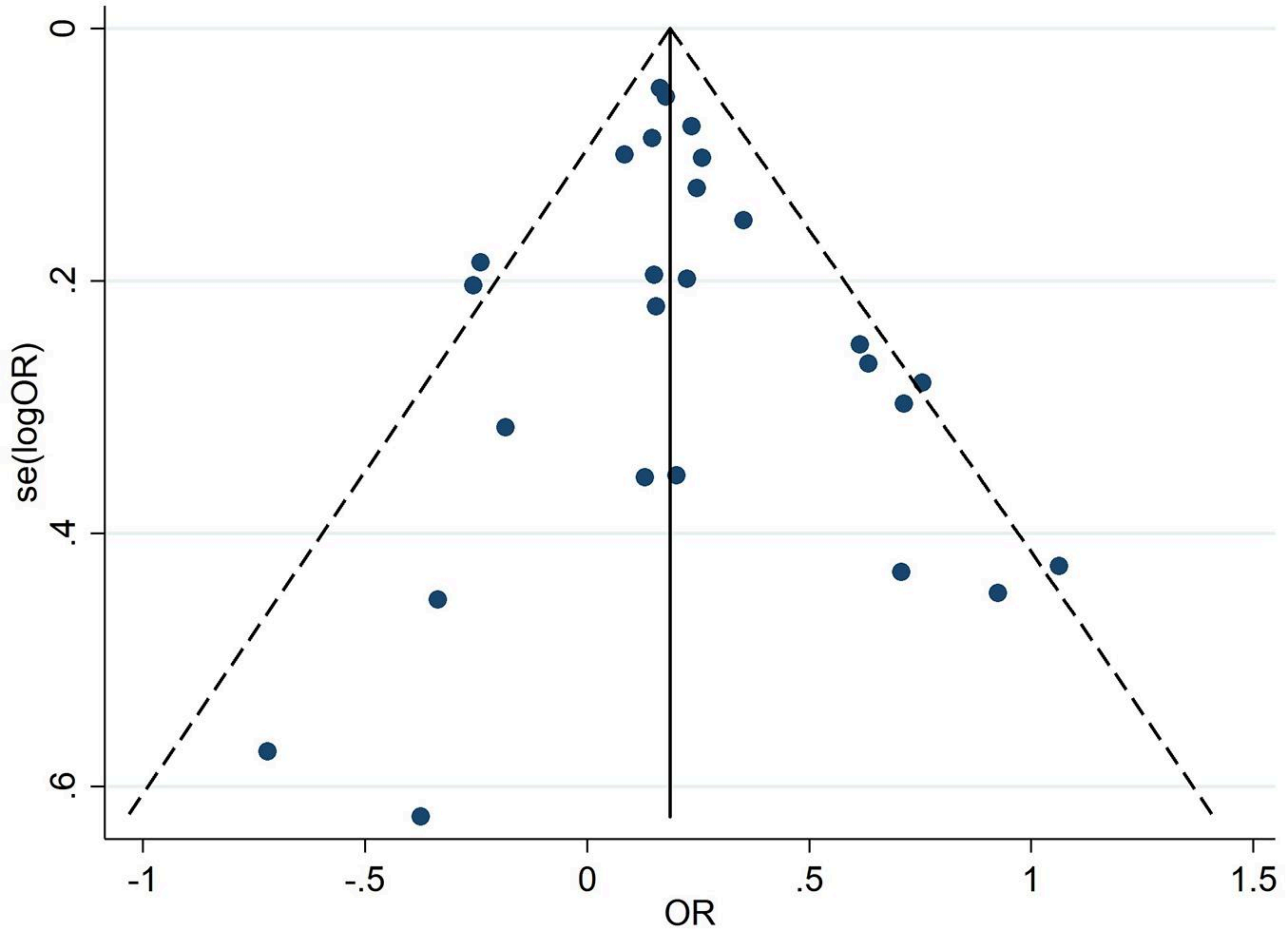
Inverted funnel plot.

The heterogeneity level of this study was 39.7%, which is acceptable. To verify the robustness of the results, we performed a sensitivity analysis using the leave-one-out method (Figure 4), where the horizontal axis represents the pool risk ratio (RR), each scatter point represents the pooled RR of the remaining studies after excluding one individual study, and the horizontal line represents the 95% CI. As shown in Figure 4, all scatter point RRs were concentrated around the original pooled results, indicating robust study results. Publication bias was evaluated using Figure 5, with each scatter point representing a single study. A small publication bias is indicated by an approximately symmetrical funnel-shaped distribution of scatter points on both sides of the effect size. The inverted funnel plot showed that scatter points were roughly symmetrically distributed around the effect axis (Figure 5), and Egger's test showed *P* = 0.436 (Table 5), indicating no publication bias and relatively reliable results. Collectively, these findings confirm the reliability of the conclusions from this meta-analysis.

**Table 5:** Egger’ test.

Std_Eff	Coef.	Std. Err.	*t*	*P*>|t|	[95% Conf. Interval]	
slope	0.156	0.051	3.07	0.005	0.051	0.261
bias	0.319	0.403	0.79	0.436	−0.512	1.150

Test of H0: no small-study effects *P* = .436.

### 3.6 Analysis of the impact of BMI on surgical complications

16 included literature reported the incidence of surgical complications in ablation procedures. Meta-analysis showed no statistically significant difference in the risk of surgical complications between obese and non-obese individuals (OR = 1.14, 95% CI:0.98–1.33, *P_value_* = 0.083), with low heterogeneity (*I^2^* = 12.3%, *P_heterogeneity_* = 0.312). Due to limited effective endpoint data, we did not compare the incidence of surgical complications across different races. This is one of the limitations of this study, and further large-scale clinical studies with are needed to validate these results.

### 3.7 Analysis of risk factors for AF recurrence

Risk factor analysis revealed that left atrial enlargement was significantly associated with the risk of AF recurrence (SMD = 0.215, *I^2^ *= 38.7%, *P_heterogeneity_* = 0.202, 95% CI: 0.03–0.40, *P_value_* = 0.021) (Table 6), with a higher LAD correlating with an increased AF recurrence risk. Other factors, including age, gender, AF duration, LVEF, and common comorbidity, showed no statistically significant association with AF recurrence.

**Table 6:** Summary of risk factors for AF recurrence.

Items	Study(n)	SMD/RR	*I^2^*(%)	*P*_*heterogeneity*_	95% CI	*P*_*value*_
Age	3	−0.066	12.1	0.321	−0.19–0.06	0.303
Duration	2	0.134	0.0	0.394	−0.01–0.27	0.059
LAD	2	0.215	38.7	0.202	0.03–0.40	0.021
LVEF	3	−0.106	51.8	0.126	−0.28–0.06	0.221
Male	3	0.970	33.2	0.224	0.90–1.05	0.479
HTN	3	0.940	63.9	0.063	0.76–1.17	0.573
DM	3	0.960	0.0	0.840	0.70–1.33	0.826

### 3.8 Meta-regression analysis

To explore potential risk factors for AF recurrence, we performed a meta-regression analysis (Table 7). With the Asian population as the reference, the association between BMI and post ablation AF recurrence was significantly weaker in European and American populations, indicating that BMI exerts a stronger impact on AF recurrence in Asians. This is consistent with the subgroup analysis results, further confirming that racial differences are a significant factor contributing to effect sizes heterogeneity among the included studies (*coef*: −.729, *P* = 0.033). Other variables, including publication year, sample size, study design, ablation strategy, and follow-up duration, had no statistically significant impact on effect size heterogeneity.

**Table 7:** Meta-regression analysis.

Item	Coef.	Std.Err.	*t*	*P*>|t|	95%CI	
					Low	Up
Publication year	−0.469	0.478	−0.98	0.339	−1.470	0.532
Sample size	0.359	0.478	0.75	0.462	−0.641	1.360
Design	0.900	0.530	1.70	0.106	−0.211	2.010
Race	−0.729	0.316	−2.31	0.033	−1.391	−0.067
Ablation strategy	0.161	0.270	0.60	0.558	−0.404	0.726
Follow-up	−0.231	0.701	−0.33	0.745	−1.698	1.236
_cons	−1.665	1.251	−1.33	0.199	−4.284	0.954

_cons, constant term in regression analysis; Coef., Regression coefficient; Std.Err., Standard error; *P*>|t|, The *P*-value of the test; 95% CI, 95% CI of the regression coefficient; *P* < 0.05 indicate a significant impact of the independent variable on the heterogeneity of the effect size.

## 4 Discussion

Based on available cohort study evidence, obese patients had an increased risk of AF recurrence post ablation. Enlarged LAD may be a potential pathological mechanism underlying AF recurrence. These results provide new evidence-based support for incorporating obesity into the comprehensive evaluation system for ablation therapy. Notably, this study is the first to identify significant racial differences in the risk of AF recurrence post ablation. The AF recurrence risk was as high as 37% in Asians (*P* < 0.001), compared with 9% in Europeans and 14% in North Americans (*P* < 0.001).

Through a large sample meta-analysis of 35,237 cases, our findings quantitatively confirmed the linear positive correlation between elevated BMI and increased risk of AF recurrence post ablation, which is highly consistent with the core basis of the NHS clinical commissioning policy. The NHS has set BMI>40 kg/m^2^ as a strict exclusion criterion for ablation and requires patients with BMI 35–40 kg/m^2^ to lose at least 10% weight before surgery (38). The core basis for this is the negative impact of obesity on ablation efficacy. Our study provides important evidence-based supplementation to this guideline and further confirms the central role of weight management in pre-ablation evaluation of AF. Additionally, this study identified racial heterogeneity in the impact of BMI on the recurrence of AF post ablation. The risk of postoperative recurrence in obese Asian populations is significantly higher than that in European and American populations. This provides an important basis for adjusting the application of Western guidelines such as NHS in Asia. Health systems in Asian countries may need to further tighten BMI indicators, appropriately lower preoperative weight loss thresholds, and increase weight loss targets to more effectively reduce the risk of recurrence. Finally, based on the NHS guidelines and our study results, we recommend incorporating BMI into global pre-ablation AF stratification assessment to develop personalized intervention strategies for patients with different BMI stratification. Racial differences should be fully considered when formulating relevant guidelines to avoid the direct application of European and American standards. Regions such as Asia should develop appropriate BMI stratification intervention strategies based on local data, and integrate weight management into the entire diagnosis and treatment pathway to enhance the long-term efficacy of ablation therapy through multidisciplinary collaboration.

The results of this study are also consistent with previous research, confirming that obesity significantly increases the risk of AF recurrence post ablation (39–42). Wong et al. found that for every 5-units increase in BMI, the recurrence risk of AF post ablation rises by 13% (22). Liu F et al. identified a borderline positive linear dose-response relationship between BMI and AF recurrence risk, indicating that obesity is a novel risk factor independent of traditional risk factors (43). Middeldorp E et al. reported that weight loss is significantly associated with reduced AF burden. When weight loss is ≥10%, 86% of patients can free from AF without ablation, and 37.5% of patients can maintain sinus rhythm with a single time ablation (44). Current guidelines also recommend a weight loss targets of >10%, and suggest that patients with BMI <27 kg/m^2^ can improve the prognosis of AF ablation through lifestyle modifications (45).

Gruberg et al. found that non-obese patients have a higher risk of postoperative complications than obese patients, which may be associated with a higher incidence of non-cardiovascular diseases in non-obese populations, particularly lean patients (46). However, another large cohort study showed no significant difference in surgical complications risk between obese and non-obese populations (47, 48), and that BMI cannot serve as a predictive indicator for surgical complications post-ablation (49). Our findings are consistent with this conclusion, with no statistical differences in surgical complication risk between obese and non-obese individuals. Due to limited effective endpoint data, this study did not perform stratified analysis of surgical complications, which is one of the limitations of our research. Future large-sample clinical studies are needed to further clarify the correlation between obesity and surgical complications risk post-ablation.

## 5 Conclusion

Our findings confirm a linear positive correlation between BMI and the risk of AF recurrence post PVI. An enlarged left atrial diameter may be one potential mechanisms underlying AF recurrence. Furthermore, significant racial disparities were identified in AF recurrence risk, with Asian patients showing a significantly higher recurrence risk than European and American patients.

## Data Availability

The raw data supporting the conclusions of this article will be made available by the authors, without undue reservation.
